# Killer Peptide-Containing Polyelectrolytic Nanocomplexes to Fight *Toxoplasma gondii* Infection

**DOI:** 10.3390/pharmaceutics17081075

**Published:** 2025-08-20

**Authors:** Arianna Bucella, Manuela Semeraro, Laura Giovati, Lorenza Artesani, Ruggero Bettini, Annalisa Bianchera, Alice Vismarra

**Affiliations:** 1 Department of Food and Drug, University of Parma, Parco Area delle Scienze 27/a Parma, 43124 Parma, Italy; arianna.bucella@unipr.it (A.B.); ruggero.bettini@unipr.it (R.B.); 2 Department of Veterinary Science, University of Parma, Strada del Taglio 10, 43126 Parma, Italy; manuela.semeraro@unipr.it; 3 Department of Medicine and Surgery, University of Parma, Via Volturno 39, 43125 Parma, Italy; laura.giovati@unipr.it (L.G.); lorenza.artesani@unipr.it (L.A.); 4 Microbiome Research Hub, University of Parma, 43124 Parma, Italy; 5 Biopharmanet-TEC, University of Parma, 43124 Parma, Italy

**Keywords:** *T. gondii*, nanoparticle, killer peptide, hyaluronate, chitosan

## Abstract

**Background/Objectives:** Toxoplasmosis, a zoonotic disease caused by *Toxoplasma gondii*, typically is asymptomatic in immunocompetent individuals but causes severe complications in immunocompromised subjects and during pregnancy. Current treatments such as pyrimethamine and sulfadiazine are effective for acute infections but cannot eliminate encysted bradyzoites and have significant side effects. The antimicrobial killer peptide (KP) has interesting therapeutic potential, but its intracellular delivery is challenging; hyaluronate-based nanoparticles loaded with KP (KP-NPs) were evaluated to target *T. gondii*-infected cells that overexpress CD44. **Methods:** KP-NPs made of chitosan and hyaluronate were produced by microfluidics and were characterized for size, surface charge, encapsulation efficiency, and stability under stress conditions. After excluding their toxicity, their activity was tested in vitro against *Candida albicans* and *T. gondii* as free tachyzoite or in infected human foreskin fibroblasts (HFFs). **Results**: KP was efficiently encapsulated in nanoparticles and protected from harsh acidic conditions at high temperature. Preliminary in vitro testing against *C. albicans* showed that, at the lowest candidacidal concentration of KP (2.5 μg/mL), KP-NPs killed 90.97% of yeast cells. KP itself proved to be non-toxic for HFFs as host cells and effective against *T. gondii*. Comparable results were obtained for KP-NPs and blank nanoparticles (BLK-NPs), with no observed toxicity to host cells, confirming that encapsulation did not alter peptide efficacy. The parasiticidal effect of KP alone, as well as KP-NPs at 250 µg/mL and BLK-NPs, was confirmed through tests on free *T. gondii* tachyzoites. Reduction rates for the number of infected cells ranged from 66% to 90% with respect to control, while the reduction in the number of intracellular tachyzoites ranged from 66% to 80%. Interestingly, KP alone was not effective against intracellular tachyzoite, while KP-NPs maintained an efficacy comparable to the extracellular model, suggesting that particles helped the internalization of the peptide. **Conclusions:** Encapsulation of KP into hyaluronate/chitosan nanoparticles does not alter its activity and improves its efficacy against the intracellular parasite. Notably, BLK-NPs appeared to exhibit efficacy against the parasite on its own, without the presence of KP.

## 1. Introduction

Toxoplasmosis is a zoonotic disease caused by *Toxoplasma gondii,* an intracellular protozoan parasite. Humans can acquire the infection through the ingestion of undercooked or raw meat containing cysts or by consuming food and water contaminated with sporulated oocysts [1]. Upon entering the human host, *T. gondii* can exist in the following two distinct forms: the rapidly replicating tachyzoite, which attaches to host cells by means of lectins and secretory proteins on its surface [2,3], and the quiescent bradyzoite, which encysts preferentially in brain tissue and skeletal muscle as a defence mechanism against immune responses, where this latent form can persist for a lifetime. However, in immunocompromised patients, the infection can reactivate, leading to severe neurological complications. Moreover, primary infection during pregnancy poses a significant risk, as the parasite can cross the placenta and cause congenital toxoplasmosis, potentially resulting in severe foetal complications [4]. In immunocompetent individuals, infection is typically asymptomatic or presents with mild flu-like symptoms, although, in the recent years, a high incidence of ocular toxoplasmosis (chorioretinitis) has been reported in immunocompetent individuals as well as congenitally infected infants in Africa and South America, often caused by atypical strains of *T. gondii* [5,6]. Finally, chronic toxoplasmosis has been shown to impair neuronal function and alter neurotransmitter levels in mice and may also be linked to conditions such as schizophrenia and bipolar disorder in humans [7,8,9].

The primary therapy for toxoplasmosis typically involves the combined use of pyrimethamine and sulfadiazine, which work synergistically by inhibiting two distinct steps in folate metabolism. Alternative regimens may include pyrimethamine in combination with clindamycin, azithromycin, or atovaquone (which suffers from poor absorption, limited membrane permeability, and inadequate oral bioavailability [10]), as well as trimethoprim with sulfamethoxazole. Despite their effectiveness, these treatments are frequently associated with toxicity and undesirable side effects, such as hematologic toxicity or allergic reactions and are ineffective against the chronic stage of the disease. In immunocompetent individuals experiencing persistent or severe symptoms, pyrimethamine-sulfadiazine is generally administered for 4 to 6 weeks. In contrast, immunocompromised patients require ongoing maintenance therapy following the initial 6-week course, due to the inability of current drugs to eradicate tissue cysts. The prolonged treatment duration, limited curative potential, and high incidence of side effects underscore the urgent need for more effective and better-tolerated anti-*Toxoplasma* therapies [11].

Some antimicrobial peptides (AMPs) have emerged as promising candidates for fighting *T. gondii* due to their ability to disrupt cellular membranes. One such peptide, killer peptide (KP), is a decapeptide (sequence: AKVTMTCSAS) derived from an anti-idiotypic antibody that mimics the yeast killer exotoxin from *Pichia anomala*. The functional unit of KP is its dimeric form, which derives from the formation of disulfide bridges between cysteines 7 in nonreducing conditions. KP has demonstrated efficacy against bacteria, yeasts, such as *Candida albicans*, both in vitro and in vivo [12], and protozoan parasites, including *T. gondii* without significant toxicity to Vero cells, used as host cell model [13]. The proposed mechanism of candidacidal action of KP involves interactions with β-glucans that catalyse the progressive self-assembly of dimers in antiparallel β-sheet eventually leading, at high concentrations, to the formation of physical gels of the peptide, which can prolong its antimicrobial effect in vivo [14]. In *T. gondii*, KP is supposed to trigger an apoptosis-like cell death, as witnessed by phosphatidylserine externalization, the dissipation of mitochondrial potential, and ultrastructural changes observed in tachyzoites [13]. Moreover, KP exhibits immunomodulatory properties [15].

Despite its therapeutic potential, intracellular delivery of peptides such as KP remains a challenging issue due to their relatively large molecular weight and high hydrophilicity. Nevertheless, targeted delivery to infected cells is a fundamental requirement to enhance therapeutic efficacy. Notably, *T. gondii* infection induces the overexpression of hyaluronan receptors, particularly CD44 and ICAM-1, in monocytes, T cells, and B cells [16,17], leading to increased adhesion of hyaluronic acid [18] and promoting the spreading of the parasite in the organism. From a therapeutic point of view, this phenomenon represents a strategic opportunity for targeted drug delivery, as already exploited in other pathologies, such as solid tumours over-expressing CD44 [19]. To exploit this internalization pathway, we propose a novel KP delivery system utilizing hyaluronate-based nanoparticles. Indeed, polysaccharidic nanoparticles offer a promising drug delivery opportunity [20] by protecting therapeutic molecules from degradation, enhancing cellular uptake, and improving bioavailability. The formulation approach was based on the formation of polyelectrolyte nanocomplexes between hyaluronate, a polyanion, and a polycationic natural polymer, chitosan, which also possesses a weak antimicrobial activity that could positively contribute to the eradication of *T. gondii* infection.

## 2. Materials and Methods

### 2.1. Materials

Killer peptide (AKVTMTCSAS, MW: 998.2, isoelectric point: 8.97) was acquired from Thermo Scientific (Rockford, IL, USA). Sodium hyaluronate (Hyal, MW: 15–30 kDa) was obtained from Contipro (Praha, Czech Republic). Chitosan (Chito, MW: 66 kDa, degree of deacetylation: 96%) was from Primex (Siglufjordur, Iceland). Acetic acid was from Merck. Ultrapure (UP) water was obtained by inverse osmosis using an Arium Comfort^®^ system by Sartorius (Gottingen, Germany). Solvents and reagents were from Avantor, VWR, unless otherwise specified. Resazurin and chlorophenol red-β-D-galactopyranoside (CPRG) were purchased from Merck-Sigma Aldrich (Milan, Italy).

### 2.2. Production of KP Loaded Nanoparticles by Microfluidics

Before formulation, peptide solubility and gelation properties were evaluated by dispersing the powder in different solvents, namely ultrapure water, phosphate-buffered saline, ethanol, methanol, or isopropanol, to evaluate its solubility and gelation properties and select the more appropriate formulative approach by microfluidics.

Sodium hyaluronate was dissolved in UP water to a final concentration of 70 µg/mL. Chitosan was dissolved at the concentration of 410 µg/mL by prolonged stirring in 0.5% *v*/*v* acetic acid aqueous solution; then, KP was added to chitosan solution at a concentration of 3.5 mg/mL. Chito/Hyal nanoparticles were prepared with an Elveflow microfluidic system (Elvesys, Paris, France) composed of an OB1 pump equipped with two flow sensors, MFS3 and MFS4, managed by the Elveflow microfluidic software (version 03_07_03). The Chito/KP solution was introduced in the reservoir connected to MFS3, while the solution of Hyal was introduced in the reservoir connected to MFS4. The two solutions were mixed in a zeonor Herringbone mixer (Microfluidic ChipShop, Jena, Germany) at a total flow rate of 280 µL/min, with a Chito/Hyal flow rate ratio of 1:6. The resulting nanoparticles were coded as KP-NPs. Blank nanoparticles (BLK-NPs) were prepared as negative control, using the same procedure except for the addition of KP to chitosan solution.

### 2.3. Characterization of NPs by Dynamic Light Scattering (DLS) and Measurement of Z-Potential

The size distribution of NPs was determined by dynamic light scattering, using a Nanozetasizer ZS (Malvern Panalitycal Ltd., Malvern, UK), operating with a laser source of He–Ne (λ = 633 nm and *p* = 5 mW). Three repeat measurements of each sample were made, using backscatter detection (θ = 173°) at 25 °C. The nanoparticles were measured without dilution by placing them in polystyrene cuvettes (Sarstedt AG, Nümbrecht, Germany). The size of the nanoparticles was expressed as average size and standard deviation of the main population detected by intensity (n = 3).

The same instrument was used to assess the Z-potential of nanoparticles, by introducing the formulation in a DTS1070 cuvette (Malvern Panalitycal Ltd., Malvern, UK).

### 2.4. Determination of Nanoparticles Composition and Encapsulation Efficiency

The amount of encapsulated KP, chitosan, and hyaluronate involved in the formation of nanoparticles were estimated as a percentage difference between the nominal amount of each component and their residual in solution. To this aim, the nanosuspensions were centrifuged at 20,000× *g* for 45 min (Neya 16R, REMI, Mumbai, India). The concentration of KP in the supernatant was quantified by Micro BCA^TM^ Protein assay kit (Thermo Scientific, Rockford, IL, USA) as per manufacturer’s instructions, using solutions of KP in the range between 25 and 500 µg/mL as reference standards.

The amount of chitosan involved in the formation of nanoparticles was determined by a colorimetric method using Reactive red 4 (MP Biomedicals, Eschwege, Germany) as described by Muzzarelli [21]. Briefly, Reactive red 4 was dissolved in ultrapure water at a concentration of 1.5 mg/mL. A buffer was prepared by mixing 81 mL of a solution containing 7.5 mg/mL glycine and 5.8 mg/mL of NaCl with 19 mL of an aqueous solution of HCl 0.1 M, then the pH was brought to 3.2 with NaOH. Finally, the working solution was prepared by mixing 5 mL of Reactive red 4 solution with 95 mL of the buffer. The quantitation of chitosan in the supernatant was performed by mixing 90 µL of the sample with 900 µL of Reactive red 4 working solution and by reading the absorbance at 572 nm. The amount of chitosan in solution was determined with respect to a reference curve built from solutions of chitosan at known concentrations in the range 5–500 µg/mL. The calibration line equation was absorbance = 0.0016 [concentration] − 0.0059, r^2^: 0.99, while the limit of detection and of quantification resulted 8 and 24 µg/mL, respectively.

The amount of hyaluronate involved in the complex was estimated by quantifying its content in the supernatant by a turbidimetric method based on the use of cetyltrimethylammonium bromide (CTAB) and adapted from Oueslati et al. [22]. Briefly, CTAB was dissolved in 2% (*w*/*v*) NaOH solution at a concentration of 25 mg/mL. Solutions of hyaluronate were prepared in water and then diluted to obtain the standard concentrations used for the preparation of the calibration line in the range of 10–100 µg/mL. For the test, 100 µL of supernatants or HA standard solution were introduced in a 96-well plate and incubated for 15 min at 37 °C. Afterwards, 100 µL of CTAB solution were added and incubated for 10 min at 37 °C. After 20 s shaking, absorbance at 600 nm was measured, and the concentration of unknown samples was estimated with respect to the calibration line. No interference was detected in the selected linearity range in the presence of chitosan.

### 2.5. Stability of Nanoparticles and Encapsulated KP

NP-KPs were stored at 4 °C, 25 °C, or 37 °C for 3 days. Size distribution, KP, chitosan, and hyaluronate content were checked daily to detect any variation associated to storage conditions.

Moreover, KP-NPs were exposed to harsh conditions, to assess the ability of the formulation to protect KP from environmental degradation. In detail, 250 µL of KP-NPs (KP concentration: 0.5 mg/mL) was diluted with 750 µL of 0.07% *v*/*v* acetic acid (as in the original formulation), 0.13 M HCl, or 0.13 M NaOH to have a final concentration of the peptide of 125 µg/mL in 0.07% *v*/*v* acetic acid, 0.1 N HCl, or 0.1 N NaOH, respectively. These three samples were exposed to high temperatures (60 °C) for 2 h, then particle size and KP content were evaluated as described above.

### 2.6. In Vitro Testing on Candida Albicans

The first in vitro tests, after the definition of the parameters necessary for the proper formation of KP nanoparticles, were initially conducted on the *C. albicans* model system, against which the antimicrobial activity of KP has been extensively characterized [14,23], and which is certainly easier to culture compared to *T. gondii*.

The first experiments aimed to highlight the difference in activity between free KP and formulated KP. To this purpose, the activity against *C. albicans* was evaluated by colony forming unit (CFU) assays, as previously described, with minor modifications [24]. Briefly, fungal cells were cultured on Sabouraud dextrose agar (SDA) plates at 37 °C for 24 h, then approximately 500 viable yeast cells were incubated at 37 °C in the absence (control) or presence of free and encapsulated KP diluted to the previously determined KP’s minimal candidacidal concentration (2.5 μg/mL) in 100 μL of sterile distilled water. BLK-NPs were used as negative control. Fungal cells in water served as growth control. After 6 h, fungal suspensions were plated on SDA, and CFU counts were performed after further incubation at 37 °C for 24 h. Percent killing was calculated relative to growth controls. Each assay was conducted in triplicate, with three independent experiments per condition.

### 2.7. In Vitro Toxicity and Efficacy Tests on the Target Parasite Toxoplasma Gondii and Infected HFF Cells

#### 2.7.1. Resazurin Reduction Assay (Alamar Blue)

Before assessing the parasiticidal activity of KP-NPs against *T. gondii*, the safety of all formulations and their components was verified on Human Foreskin Fibroblasts (HFFs) using the Resazurin reduction assay (alamarBlue). For the resazurin reduction assay, HFFs were seeded in 96-well plates at a density of 5 × 10^3^ cells/well and cultured for 72 h at 37 °C/5% CO_2_. Afterwards, compounds (KP alone, KP-NPs, and BLK-NPs) were diluted in Dulbecco’s Modified Eagle Medium (DMEM) complemented with 10% FBS to a concentration of KP of 250 µg/mL (or corresponding number of BLK-NPs) and added to HFFs cultures at 80% confluency (200 μL/well). Negative controls (untreated) were prepared in DMEM + 10% FBS. KP alone was tested on HFF cells at concentrations of 428, 214, 107, 54, 26, 13, and 6 µg/mL. All components of the nanoparticles (Hyal, Chito, and acetic acid) were also tested individually starting from their concentrated aqueous solutions diluted in DMEM + 10% FBS at the same final concentrations at which they were present in the formulations, namely 60 µg/mL for each of the two polymers and 0.07% *v*/*v* for acetic acid. The medium was discarded and substituted with test compounds for 48 h, then viability of HFFs was determined by Resazurin reduction assay (alamarBlue) [25]. Each condition was tested in six wells, and each experiment was conducted in three independent assays. Briefly, a stock of resazurin concentrated at 2 mg/mL was prepared and stored at 4 °C until use. Cells were washed three times with phosphate saline buffer (PBS) 1×, then resazurin was diluted in PBS into each well to reach the final concentration of 10 μg/mL. Plates fluorescence was read (excitation 530 nm, emission 590 nm wavelength) using a VICTOR^®^ Nivo™ Multimode Microplate Reader (PerkinElmer, Waltham, MA, USA). The output data were expressed in relative fluorescence units (RFUs) and used to compare the fluorescence intensity detected at the level of the well of the treated samples against the negative controls. Fluorescence-reported values were evaluated through specific steps, as follows: initially, two readings were taken, one representing time zero (T0) when the resazurin was added, and a second reading taken three hours later. This was followed by calculations using Microsoft Excel^®^ software package (Microsoft, Redmond, WA, USA). The values obtained from the two readings were subtracted from each other, and from these, the respective averages of each group were calculated. The standard deviation and the percent (normalized) fluorescence were calculated as a ratio between the mean of the treatments and the mean of the negative control (DMEM + 10% FBS), multiplied by 100. Half-maximal cytotoxic concentration (CC_50_) values were calculated after the logit-log-transformation of relative growth and subsequent regression analysis. The CC_50_ value corresponds to the minimum concentration of a compound capable of inhibiting 50% of the cell growth.

#### 2.7.2. Toxo-β-Gal Assay

*T. gondii* β-gal-RH (Type I) is a genetically modified strain constitutively expressing β-galactosidase under the control of the promoter SAG1 (constitutive promoter). Susceptibility of *T. gondii* β-gal to the tested compounds was assessed by determining IC_50_ values based on β-galactosidase assay, as described by Păunescu et al. [26] Briefly, HFFs (5 × 10^3^/well) were seeded into 96-well plates, and once confluent, 200 µL of a suspension of parasites prepared in DMEM + 10% FBS at the concentration of 7.5 × 10^4^/mL was added to each well resulting in 1.5 × 10^4^ parasites per well.

The IC_50_ was determined in a pre-infection in vitro model (effect on the extracellular parasites) and in a post-infection in vitro model (effect on the intracellular parasites), as follows: in the first case, test compounds (KP alone, KP-NPs, and BLK-NPs) were prepared in DMEM complemented with 10% FBS (KP concentration: 250 µg/mL) and added simultaneously to infection of host cells; in the second case, 3 h post-infection (the time needed for parasites to invade cells), the medium with the parasites was removed, cells were washed three times to eliminate extracellular and non-internalized parasites, then the medium containing the compounds was added [27,28]. A negative control was prepared in DMEM + 10% FBS; all components of formulations, namely hyaluronate, chitosan, and acetic acid, were tested as well at the same concentrations present in nanoparticles. Plates were incubated for 48 h 37 °C, 5% CO_2_, then cultures were washed once with 200 μL/well of PBS 1×. Subsequently, 90 μL per well of Triton-X at 0.05% were added to the plates to lyse cells and release β-galactosidase enzyme. In total, 10 μL of 5 mM chlorophenol red-β-D-galactopyranoside (CPRG) was added to each well, and the reaction was monitored until saturation (usually reached in 30 min) by repeatedly measuring absorbance at 570 nm. The values obtained from the initial readings were subtracted from the respective final readings, and from these, the averages of each group were calculated. This was followed by calculation of the standard deviation and the calculation of percentage (normalized) absorbance, which was obtained from a ratio between the mean of the treatments and the mean of the negative control (DMEM 10% FBS), multiplied by 100. IC_50_ values were calculated after the logit-log-transformation of relative growth and subsequent regression analysis using Microsoft Excel^®^ software package (Microsoft, Redmond, WA, USA). The IC_50_ value corresponds to the minimum concentration of a compound capable of inhibiting 50% of parasites.

#### 2.7.3. In Vitro Testing on the Free Form of the Parasite (Tachyzoites)

Free tachyzoites were cultured under standard conditions, subsequently purified, counted, and exposed to the substances of interest (KP, KP-NP, and BLK-NPs) for 2 h to determine whether the tachyzoites were still capable of penetrating host cells, infecting them, and multiplying without interference. A parasite-to-cell ratio of 5:1 was used. To perform these experiments, we developed a specific protocol involving seedings on coverslips, followed by staining, scanning, and counting. Before scanning, coverslips were mounted on glass slides. Each condition was repeated in duplicate (two slides per condition), and the experiment was repeated three times. The parameters evaluated, considering 100 cells from four different quadrants, were as follows

- Number of infected cells;
- Number of intracellular parasites.

The counting was performed after scanning the slides using a Research Light Scanner VS200 (Olympus, Tokyo, Japan). Three different operators conducted the counts, repeating them in different quadrants three times for a total of 300 cells counted per slide. The average of the three counts (for each slide/condition) was then calculated, along with the standard deviation.

Additionally, the same experiment was performed in duplicate to extract DNA for subsequent quantification of the parasite using real-time PCR CFX96™ Biorad^®^ (version 3.1) (without using coverslips in the wells). The protocol by Santoro et al. [29], amplifying the target B1 gene using Taqman technology, was optimized by defining the appropriate annealing temperature and amplification cycles. A standard curve for quantification was generated using *T. gondii* RH-WT grown in HFF cells, to ensure consistency in the results. DNA was extracted with the commercial kit Invitrogen PureLink™ Genomic DNA Mini Kit (Thermo Fisher Scientific, Waltham, Massachusetts, USA) from infected cells (control/treatment) 48 h after seeding, and it was used for amplification of the B1 gene.

The resulting product was loaded onto an agarose gel, and the corresponding band was excised for DNA quantification (12.3 ng/µL). From this, the number of copies per µL (1.1 × 10^11^) was calculated using Copy Number Calculator (accessed on 11 April 2025)) [30], and a standard curve was generated via serial dilutions (starting from a concentration of 10^8^ copies of DNA/µL down to 10^2^ copies of DNA/µL). Each condition was tested in four wells; three independent experiments were conducted.

### 2.8. Statistical Analyses

Peptide and nanoparticle activity on *C. albicans* was evaluated with one-way ANOVA followed by a *post hoc* Tukey’s multiple comparisons test, using GraphPad Prism^®^ version 8.0.2 Software (GraphPad Software, San Diego, CA, USA). The activity on *T. gondii* was evaluated with Student’s T test and ANOVA calculated with GraphPad Prism^®^ and with non-linear regression using Microsoft Excel^®^ software package (Microsoft, Redmond, WA, USA). A *p*-value < 0.05 was considered statistically significant.

## 3. Results

### 3.1. KP Solubility in Different Solvents

The solubility of KP and the flowability of the resulting solution are critical elements for microfluidic processing, since viscous solutions cannot be used, as they alter laminar flow conditions or clog the chip’s channels. For this reason, common solvents were screened to select those providing the highest solubility, to prevent spontaneous precipitation of the peptide, without neglecting their acceptability from the toxicological point of view. The peptide was practically insoluble in isopropanol and acetone. As previously reported in the literature [23], the peptide showed a good solubility in DMSO (20 mg/mL) with no apparent changes in time, even after prolonged storage; solubility in methanol was verified up to 2 mg/mL. Anyway, these two solvents have a poor toxicity profile and were for this reason excluded from further tests. On the other hand, KP is soluble in ethanol at least up to 2 mg/mL, but its dissolution at this concentration is followed by an almost immediate transition, leading to the formation of a thick gel, which was not suitable for formulation by microfluidics. This behaviour shares some similarities with what happens in water; actually, KP assumes a random coil conformation right after its dissolution but forms aggregates that acquire a β-sheet conformation with time, leading to a progressive transition from colloidal solution to highly viscous hydrogel. The kinetic of the process was concentration-dependent, being almost instantaneous at the concentration of 5 mg/mL, while at a concentration of 3.5 mg/mL, the sol–gel transition occurred over the course of two days at room temperature. Unexpectedly, KP was practically insoluble in PBS, leading to the exclusion of this buffer from formulation steps. These preliminary observations were useful for implementing the formulation strategy and for determining the optimal KP concentration to be used in the system. Thus, we decided to work with aqueous KP solutions up to a maximum concentration of 3.5 mg/mL, prepared on the same day, as it was submitted to the formulation process.

### 3.2. Production, Characterization, and Stability of NPs

Nanoparticles loaded with KP were prepared by microfluidics to obtain polyelectrolytic complexes based on chitosan and hyaluronate. The peptide was included in chitosan solution at a concentration of 3.5 mg/mL. At total of 5 mL of solution was collected for each preparation batch. The use of microfluidic provided consistency in the characteristics of resulting particles, in terms of size and encapsulation efficiency of the peptide. The average size and distribution of particles, Z-potential, and encapsulation efficiency of KP (EE %) are reported in Table 1.

The size of blank nanoparticles was lower than loaded ones, indicating that an interaction occurred between polymers and peptide, as also shown by satisfactory encapsulation efficiency of KP. The negative Z-potential of nanoparticles suggests that hyaluronate was located on the external part of the nanoparticles, namely in the right place for potentially driving their cell internalization through binding to the CD44 receptor. The KP-NPs were stored at 4, 25, and 37 °C for 3 days to assess any variations in particle size, KP, hyaluronate, or chitosan content that may occur during the execution of in vitro tests. Under each condition, the parameters were compared to those obtained with the freshly prepared formulation and are reported in Table 2.

KP-NPs showed a substantial particle size stability over the 3-day period in all storage conditions, with a slight tendency to reduction at higher temperatures. KP, hyaluronate, and chitosan content did not change significantly over the 3-day period under all storage conditions, suggesting that no rearrangement of the complexes nor leakage of the peptide occurred.

To further assess the stability of KP-NPs and their potential protective effect on KP, both free KP and KP-NPs were subjected to harsh stress conditions, namely high temperature (60 °C) and strong acidic or alkaline environment (0.1 M HCl or NaOH) for two hours. Free KP demonstrated substantial stability, with full recovery under high temperature and acidic conditions, while approximately 20% of peptide was degraded after treatment in alkaline conditions at 60 °C. As for nanoparticles, particle size was checked (Table 3), and the amount of KP released quantified. No KP was detected in the supernatant from KP-NPs after treatments, suggesting that the peptide remained encapsulated within the particles and was not released, even under these extreme conditions, despite a significant variation in particle size; in particular, high temperature and alkaline conditions lead to an increase in average size and width of distribution, while acidic conditions, even at high temperature, lead to a shrinkage of the particles. These differences can be ascribed to a change in the protonation state of the two polymers at different pH levels, leading to a variation in the strength of ionic interactions between them.

### 3.3. Tests on Candida Albicans Model

**Figure 1:**
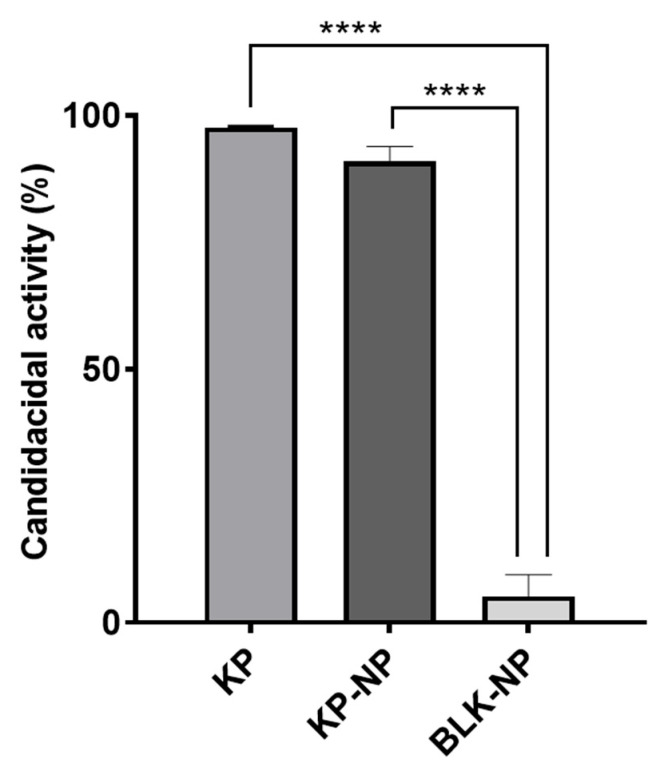
Percent candidacidal activity. The results were obtained after challenging *C. albicans* with KP at 2.5 µg/mL (minimal fungicidal concentration), KP-NPs corresponding to 2.5 µg/mL of KP, and BLK-NPs. Data are presented as mean ± SD of three independent experiments (three replicates per condition in each experiment). Statistic was made applying one-way ANOVA with Tukey *post hoc* (GraphPad Prism) (**** *p* < 0.0001).

At the minimal candidacidal concentration of KP (2.5 μg/mL), KP-NPs were able to kill 90.97% of *C. albicans* cells, showing a non-significant variation in the activity as compared to the free peptide (Figure 1). BLK-NPs did not show candidacidal activity. The results suggested that the KP-NPs’ formulation did not reduce KP activity and was, therefore, considered a promising approach for further experimentation.

### 3.4. Tests on T. gondii

#### 3.4.1. Toxicity and Efficacy on HFF Cells and *T. gondii*

Table 4 and Table 5 report the toxicity and efficacy of the components of nanoparticle formulations (used at a concentration of KP at 250 µg/mL). Results highlight that nanoparticles and HA were not toxic to host cells. Although acetic acid alone and chitosan alone (tested at the same concentrations present in nanoparticles) exhibited toxicity, the formulation of KP in HA-based nanoparticles prepared with acetic acid was not toxic. KP alone was tested on HFF cells at concentrations of 428, 214, 107, 54, 26, 13, and 6 µg/mL. At all tested concentrations, cell viability ranged from 91% to 100%, indicating that KP is not toxic to host cells, as already established [13].

**Figure 2:**
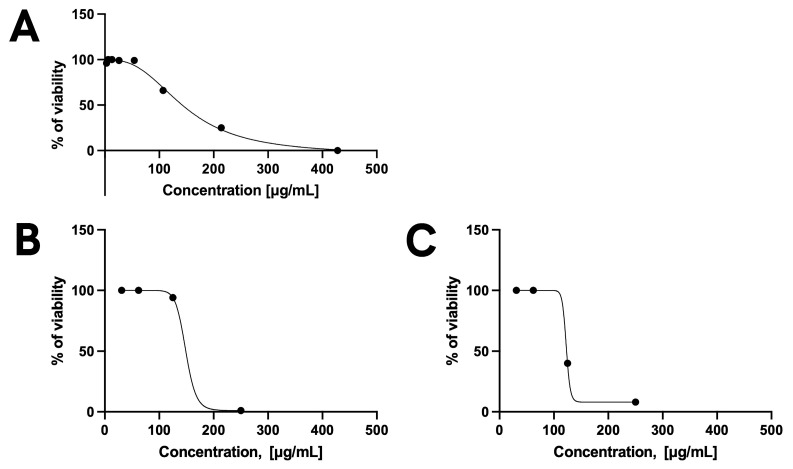
Dose–response curves of free KP against extracellular *T. gondii* (**A**), KP-NPs against extracellular *T. gondii* (**B**), and intracellular *T. gondii* (**C**). Free KPs did not show significant activity against intracellular *T. gondii*.

Nevertheless, the cells exhibited some degree of stress due to nutrient deprivation, especially during the standard 72-hour experiment. As a result, the Toxo-β-gal assay was modified to assess efficacy after 48 h rather than 72 h. The results show that KP was effective against extracellular *T. gondii* at concentrations ranging from 75 and 195 μg/mL (Figure 2A). Regarding the nanoparticles (KP-NPs), the best efficacy against extracellular *T. gondii* was achieved at concentrations between 100 and 170 μg/mL, without inducing toxicity in host cells (Figure 2B).

**Figure 3:**
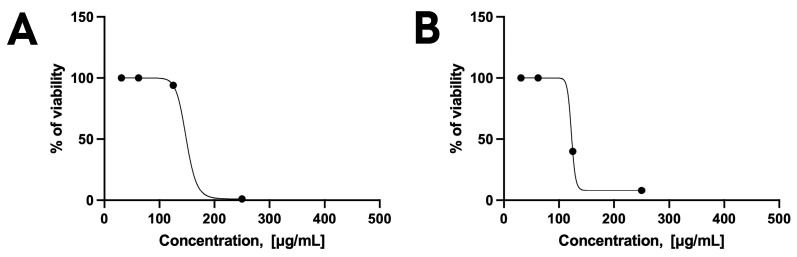
Dose–response curves of BLK-NP s against extracellular *T. gondii* (**A**) and intracellular *T. gondii* (**B**) calculated on three independent experiments (six replicates per experiment).

These findings further confirm, as previously suggested by tests on *C. albicans*, that encapsulation preserved the activity of KP. Curiously, comparable results were obtained with BLK-NPs, indicating that the nanoparticle itself may positively contribute to antiparasitic activity (Figure 3A). Most importantly, KP alone did not exhibit any efficacy against intracellular *T. gondii*, whereas both KP-NPs and BLK-NPs maintained their efficacy against the intracellular parasite (CC_50_ ranging between 109 µg/mL and 120 µg/mL) (Figure 2C and Figure 3B). This suggests that the formulation into nanoparticles favours the internalization of KP into the cells and improves its activity against the target.

Unexpectedly, sodium hyaluronate solution as well reduced parasite viability to 30% in the extracellular model and to 7% in the intracellular model, suggesting that its role could go beyond the promotion of nanoparticle internalization into host cells, by directly affecting *T. gondii* viability (Figure 3, Table 5). On these bases, the concentration of 250 µg/mL was selected for further tests to confirm the parasiticidal activity against free tachyzoites.

#### 3.4.2. Efficacy on Free Tachyzoites

**Figure 4:**
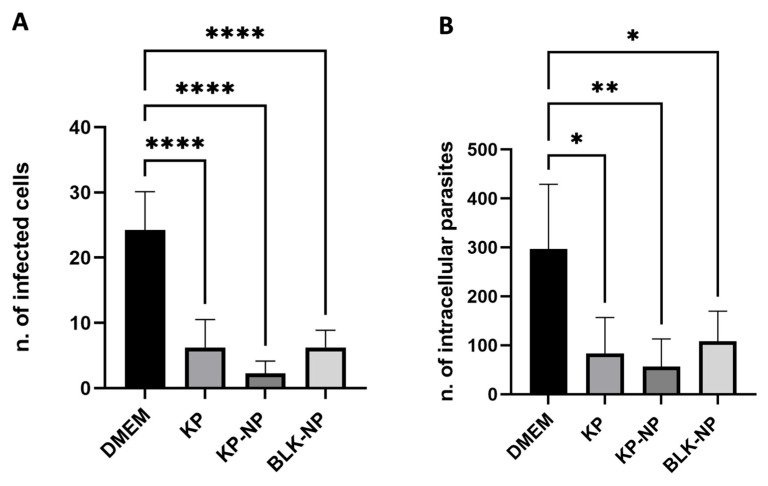
Numbers of infected cells (**A**) and intracellular parasites (**B**) after treatment with KP at 250 µg/mL, KP-NPs corresponding to 250 µg/mL of KP, and BLK-NPs. DMEM was used as negative control (infected, non-treated cells). Data are present as mean ± SD of three independent experiments conducted in duplicate (two slides per condition). For each slide, counts were obtained by three different operators using Research Slide Scanner VS200 (Olympus) and considering 100 cells from four different quadrants. Statistic was made applying one-way ANOVA with Tukey *post hoc* (GraphPad Prism). *p* values reported in figure are referred to the comparison with negative control (* *p* < 0.05; ** *p* < 0.01; **** *p* < 0.0001).

The activity of KP at 250 µg/mL was evaluated in comparison with the corresponding concentration of KP-NPs and the relevant BLK-NPs. The test on free tachyzoites confirmed the parasiticidal activity on *T. gondii* for KP alone, as well as for KP-NPs and for BLK-NPs, with reduction rates ranging from 66% to 90% (considering the number of infected cells) (Figure 4A) and from 66% to 80%, considering the number of intracellular tachyzoites (Figure 4B). *p*-values indicated a highly statistically significant difference considering both the number of infected cells and the number of intracellular tachyzoites between controls and treated slides (*p* < 0.0001), highlighting a parasiticidal effect by all conditions tested (KP alone, KP-NPs, and BLK-NPs).

**Figure 5:**
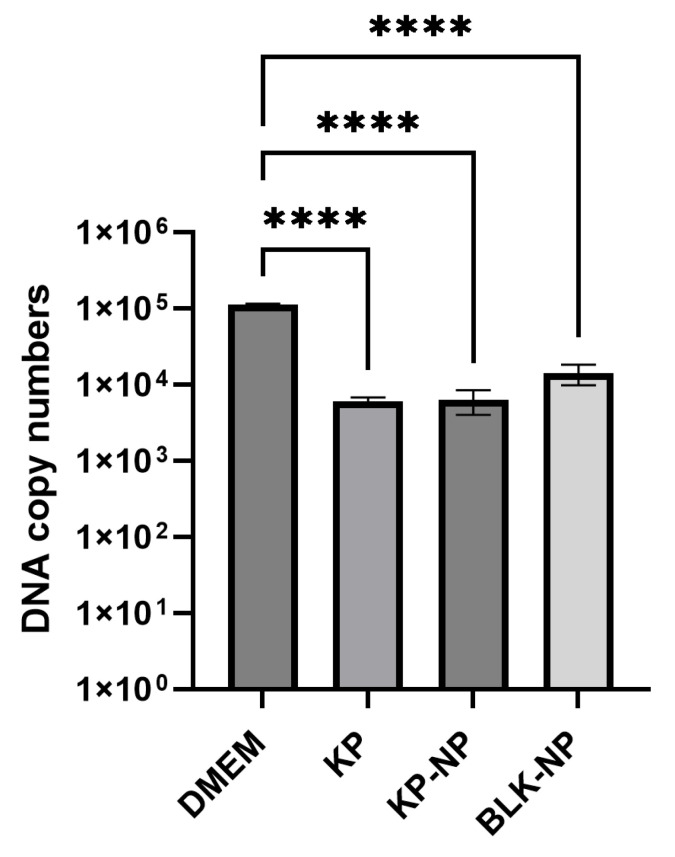
Quantification of DNA copy numbers of each treatment (KP at 250 µg/mL, KP-NPs corresponding to 250 µg/mL of KP, and BLK-NPs) compared to the control (DMEM: infected, non-treated cells) obtained via real-time PCR and expressed as Log_10_. Each condition was tested in four wells, and data are presented as mean ± SD of three independent experiments. Statistics were made applying one-way ANOVA with Tukey *post hoc* (GraphPad Prism) (**** *p* < 0.0001).

The quantification data obtained via real-time PCR also confirmed the findings from the slide counting experiments, supporting the effectiveness of the treatments in reducing parasite load. Parasite load was reduced by 94.6%, 94.4%, and 87.4% with KP, KP-NP, and BLK-NP treatment, respectively, compared to the control. The differences among the DNA copies of treated (KP 250 µg/mL; KP-NPs; BLK-NPs) and non-treated samples were statistically significant too (*p* < 0.0001) (Table 6, Figure 5).

## 4. Discussion

The therapeutic options for toxoplasmosis are still limited, with pyrimethamine and sulfadiazine being the first-choice drugs for treating acute infections. While effective, these drugs are associated with significant side effects and fail to eliminate encysted bradyzoites due to their inability to penetrate host tissue effectively. Consequently, there is an urgent need for alternative, effective, and safe therapeutic strategies targeting *T. gondii* [10,11].

In this study, we encapsulated KP, a peptide previously shown to be active against extracellular *T. gondii* [13], into polymeric nanoparticles to enhance cellular uptake and overcome the inherent challenges posed by peptides’ molecular weight and hydrophilicity. Nanoparticles can improve the therapeutic potential of drugs thanks to enhanced drug stability, efficacy, targeting specificity and tolerability, and intracellular penetration [20]. In the context of toxoplasmosis, *T. gondii-*infected cells are reported to overexpress CD44 receptors [16], which are involved in the binding and uptake of hyaluronan [31]. This offers an interesting opportunity for targeted intracellular delivery of KP by means of hyaluronan-based nanoparticles, as previously reported for other proteins [32]. To preserve the structural integrity and activity of sensitive peptides during formulation, we adopted a microfluidic technique, a mild processing method that avoids organic solvents and ensures high reproducibility, encapsulation efficiency, and scalability from minimal reagent volumes. To obtain polyelectrolytic complexes, hyaluronan was associated with chitosan [33]. This polymer was chosen for its positive charge at acidic pH as well as for its known antimicrobial and antifungal properties [34]. Intriguingly, recent studies also suggest a broad antiparasitic activity of chitosan as a raw material [35,36,37,38] and, in the form of nanoparticles, specifically against *T. gondii* [39], particularly when using low-molecular-weight chitosan or its oligomers. Chitosan could also aid targeting by interacting with the chitin binding protein of *T. gondii* [40]. Based on these observations, we selected low-molecular-weight (66 kDa) chitosan and combined it with low-molecular-weight (15–30 kDa) hyaluronate, deliberately excluding additional excipients or gelation agents, to prevent interference with KP’s activity.

Incorporation of KP into nanoparticles led to an increase in their size with respect to unloaded ones, consistent with the high estimated encapsulation efficiency. The negative Z-potential of particles indicates that hyaluronan was located on their surface, exposing the negatively charged carboxylic groups. This may facilitate CD44-mediated internalization by target cells, such as fibroblasts, used in this paper as in vitro infection model, or monocytes which are infected in vivo by *T. gondii* [16]. Notably, NPs demonstrated good physical stability in terms of the size and content of peptide and polymers for the time and temperature conditions required to complete the in vitro assays, as well as under stress conditions, suggesting that encapsulation can protect the peptide from environmental degradation. However, extended stability studies will be necessary to assess the shelf-life and ideal storage conditions for the formulation in view of a potential pharmaceutical application. Preliminary in vitro assays against *C. albicans* confirmed that the activity of KP was preserved after encapsulation, supporting progression to anti-*T. gondii* studies. Toxicological assessments of formulation components showed no adverse effects. Moreover, KP-NPs retained full biological activity against extracellular tachyzoites, at a concentration between 100 and 170 μg/mL, demonstrating efficacy comparable to that of free KP (range between 75 and 195 μg/mL). Interestingly, while free KP showed no efficacy against intracellular *T. gondii*, KP-NPs retained their activity, suggesting enhanced intracellular delivery of the peptide. It would be valuable to visualize KP internalization mediated by nanoparticles, for instance by fluorescence microscopy, using a fluorescently tagged version of the peptide. However, this strategy should be considered with caution, since any covalent modification of such a short peptide could dramatically modify its physicochemical characteristics, potentially altering its loading efficiency into nanoparticles and, most importantly, impairing its parasiticidal activity [41]. A previous in vitro study [13] showed that KP may induce an apoptosis-like cell death in *T. gondii* tachyzoites; however, an in vivo study on mice together with histological analyses might certainly clarify this point. From previous studies on yeasts, it has been hypothesized that the first step of KP killing activity is an interaction with cell-wall, glucan-like structures, which may be followed by the induction of intracellular ROS and cause a rapid decrease in mitochondrial transmembrane potential when tested as antifungal drugs, as shown by studies on peptides derived from KP (e.g., K10S, H10S) [42].

Cell wall glucans are absent in *T. gondii* tachyzoites [23], indicating that different multi-modal mechanisms may be involved against different pathogens, as was demonstrated when showing that this peptide may also act against viruses by interaction with different targets [43]. For *T. gondii*, chitosan-binding proteins or chitinase-like proteins expressed by the parasite could be involved [40], but this hypothesis needs further investigation too.

Unexpectedly, BLK-NPs revealed a comparable activity against the intracellular parasite, suggesting a possible intrinsic antiparasitic role of the nanoparticle itself. From a parasitological point of view, the fact that the nanoparticles developed here are effective against *T. gondii*, also without KP, is very intriguing and represents a novel finding. In fact, to the best of our knowledge, no literature describes a parasiticidal role of hyaluronate; while some studies have investigated the parasiticidal activity of chitosan-based nanoparticles, this was limited to the extracellular form or free tachyzoites [39]. Only a paper by Etewa et al. [44] describes mild antiparasitic activity of chitosan but to a lesser extent with respect to data shown here. Regarding the carrier, and considering the above listed limits of current treatments, if the potential of the combination of delivery system and of KP will be confirmed by further in vivo studies, this could constitute a significant step forward in the search for new effective strategies against *T. gondii,* considering that most of the compounds tested in vitro failed when used in mice in vivo [45,46]. In fact, the same delivery system could alternatively be employed as a carrier for spiramycin or pyrimethamine, potentially enabling their use even against the cystic form of the parasite. Moreover, the nanoparticulate system might allow for a lower drug dose to achieve parasiticidal activity, thereby potentially reducing side effects. The same could also be hypothesized for spiramycin, which has an IC_50_ of 218 µg/mL in vitro when used against intracellular tachyzoites of *T. gondii* [47].

BLK-NPs showed no cellular toxicity offering an advantage over other types of nanoparticles, such as silver nanoparticles, which despite their potential against *T. gondii,* have intrinsic toxicity and can accumulate in the organism [48]. The mechanism of action of this formulation remains unclear and undoubtedly deserves further investigation.

## 5. Conclusions

In this paper, we described the formulation of KP into chitosan/hyaluronate nanoparticles resulting in its improved ability to penetrate infected cells and thus to act against intracellular tachyzoites, opening promising perspectives for the treatment of chronic toxoplasmosis. Further investigation will delve into the mechanism of action of the formulation and its components, also exploring its potential use for encapsulating other anti-parasitic drugs, such as, for example, spiramycin, which can induce significant side effects when used alone. Further studies will be needed, also including, for example, bradyzoites in cell monolayers or in three-dimensional cultures, before moving on to a potential in vivo approach.

## Figures and Tables

**Table 1:** Physical characteristics and composition of blank (BLK-NP) and KP-loaded (KP-NP) nanoparticles; values are reported as mean ± standard deviation.

Formulation	Particle Size (nm)	Z-Potential (mV)	KP EE (%*w*/*w*)	Chitosan Involved (% *w*/*w*))	HA Involved (% *w*/*w*)
BLK-NPs	242 ± 81	−13.1 ± 1.6	-	70 ± 6	90.1 ± 0.9
KP-NPs	392 ± 100	−14.6 ± 1.03	76 ± 9	88 ± 4	94.6 ± 0.7

**Table 2:** Particle size, KP, and chitosan content under different storage conditions over 3-day period; values are reported as mean ± standard deviation.

Temperature (°C)	Day	Particle Size (nm)	KP Content (% *w*/*w*)	Chitosan Content (% *w*/*w*)	HA Content (% *w*/*w*)
	1	379 ± 36	63.0 ± 1.6	93.9 ± 6.3	93.7 ± 0.1
4	2	357 ± 37	62.6 ± 1.1	84.9 ± 3.6	93.4 ± 1.7
	3	384 ± 45	64.3 ± 4.1	85.7 ± 0.4	93.9 ± 1.0
	1	235 ± 29	63.4 ± 2.4	80.9 ± 8.4	90.1 ± 7.2
25	2	564 ± 108	63.5 ± 2.5	93.3 ± 8.8	93.1 ± 0.4
	3	359 ± 48	64.4 ± 4.2	93.2 ± 8.8	92.8 ± 0.4
	1	295 ± 34	63.1 ± 1.8	95.6 ± 3.8	95.7 ± 1.3
37	2	320 ± 43	62.2 ± 0.8	90.3 ± 2.9	93.9 ± 1.5
	3	302 ± 33	65.4 ± 6.4	79.9 ± 0.2	94.6 ± 0.3

**Table 3:** Size of nanoparticles after stress treatments; values are reported as mean ± standard deviation.

Treatment	Particle Size (nm)
Room temperature	404 ± 107
60 °C	734 ± 342
0.1 N HCl, 60 °C	213 ± 70
0.1 N NaOH, 60 °C	478 ± 157

**Table 4:** Toxicity of single components and nanoparticles (expressed as a percentage value of cells viability % ± standard error) on HFFs at concentrations corresponding to 250 µg/mL of formulated KP.

	Sodium Hyaluronate Solution	Acetic Acid	Chitosan Solution	BLK-NPs	KP-NPs
Viable HFFs	95% (±0.06) -> nontoxic	0% (±0.06) -> toxic	0% (±0.50) -> toxic	84% (±1.6) -> nontoxic	93% (±2.29) -> nontoxic

**Table 5:** Efficacy of single components and nanoparticles (expressed as a percentage value of parasites viability % ± standard error) on extracellular and intracellular tachyzoites, at the concentration corresponding to 250 µg/mL of formulated KP. Acetic acid and chitosan solutions were not tested as they were toxic on HFF cells (see Table 4).

	Sodium Hyaluronate Solution	BLK-NPs	KP-NPs
Viable extracellular tachyzoites	24% (±0.50) -> effective	9% (±0.38) -> effective	9% (±0.33) -> effective
Viable intracellular tachyzoites	8% (±0.20) -> effective	7% (±0.20) -> effective	8% (±0.09) -> effective

**Table 6:** Quantification data obtained via real-time PCR (calculated with the CFX96™ Biorad^®^ software) and % of parasite load reduction in treated samples compared to the control (DMEM). SQ: starting quantity.

Sample	Cq Mean	Cq Std. Dev	SQ	Log SQ	SQ Mean	SQ Std. Dev	ΔCq	FC	% Reduction Rate
DMEM CTR	24.37	0.688	48,677	4.687	111,373	4194.25			
KP	28.17	0.177	5512	3.741	6041	747.92	3.80	0.072	94.6
KP-NPs	28.17	0.530	4668	3.669	6252	2240.29	3.79	0.072	94.4
BLK-NPs	26.99	0.22	16,977	4.230	14,022	4179.11	2.62	0.163	87.4

## Data Availability

The raw data supporting the conclusions of this article will be made available by the authors on request.

## Abbreviations

The following abbreviations are used in this manuscript:

KP, Killer Peptide; KP-NPs, Killer Peptide-Nanoparticles; BLK-NPs, Blank Nanoparticles; HFF, Human Foreskin Fibroblasts, *T. gondii, Toxoplasma gondii*; *C. albicans, Candida albicans;* UP, ultrapure; CPRG, chlorophenol red-β-D-galactopyranoside; CTAB, cetyltrimethylammonium bromide; CFU, colony forming unit; SDA, Sabouraud dextrose agar; RFU, Relative Fluorescence Unit; DMEM, Dulbecco’s Modified Eagle Medium; EE, Encapsulation efficiency.
